# Examining the Cognitive Underpinnings of Functional Decline in Prodromal Alzheimer's Disease: Insights From the Details of Functions of Everyday Life (DoFEL) Scale

**DOI:** 10.1155/jare/2610700

**Published:** 2025-08-12

**Authors:** Freddie O'Donald, Clara Calia, Mario A. Parra

**Affiliations:** ^1^School of Health in Social Science, University of Edinburgh, Edinburgh, UK; ^2^Department of Neuropsychology, NHS Tayside, Dundee, UK; ^3^School of Psychological Sciences and Health, University of Strathclyde, Glasgow, UK

**Keywords:** dementia, early detection, functional assessment, mild cognitive impairment, visual short-term memory binding

## Abstract

Available assessments for early-stage Alzheimer's disease (AD) identify neuropsychological and functional impairments, which rarely correlate in the early disease stages. The ability to bind information in memory declines in preclinical AD stages. However, it is unclear whether such cognitive deficits underlie functional impairment in prodromal AD stages. This study investigates whether incorporating memory binding, a function that is a sensitive cognitive marker for early-stage AD, into a functional assessment tool can reveal the cognitive underpinnings of daily activities. The Details of Function of Everyday Life (DoFEL) scale was revised, and its latent structure was explored through principal axis factoring in a nonclinical sample (*n* = 559). Dementia professionals subsequently reviewed the revised DoFEL for content validity, followed by confirmatory factor analysis in another nonclinical sample (*n* = 135). Additionally, 49 participants with mild cognitive impairment (MCI) and 33 healthy controls completed the DoFEL, Addenbrooke's Cognitive Examination-Revised (ACE-R) and a Visual Short-Term Memory Binding Task (VSTMBT). Correlation analysis and binomial regression were used to examine the relationship between DoFEL scores and cognitive measures and to assess its ability to differentiate between healthy controls and MCI patients. The revised DoFEL showed satisfactory structural and construct validity, although some items lacked content validity. Significant negative associations were found between DoFEL scores and ACE-R (*r* = −0.66, *p* < 0.001) as well as VSTMBT (*r* = −0.52, *p*=0.003) performances. Binomial regression demonstrated the DoFEL's effectiveness in distinguishing healthy controls from MCI patients (AUC = 0.95). These findings suggest that linking memory binding with functional performance could enhance functional assessment in early-stage AD.

## 1. Introduction

Alzheimer's disease (AD) is the primary cause of dementia and is characterised by progressive neurodegenerative changes, leading to cognitive and functional decline [1, 2]. In recent years, it has been recognised that AD has an identifiable prodrome characterised by distinct clinical stages preceding significant cognitive and functional decline [2, 3]. Mild cognitive impairment (MCI) is acknowledged as an intermediary phase between normal ageing and dementia and is associated with an increased risk of AD [4, 5]. MCI is characterised by cognitive decline beyond expectation for age, with relative preservation of functional abilities [6, 7]. Efforts to identify symptom–biomarker associations indicating neurodegenerative changes in MCI that predict conversion to AD are a key focus within the field [8, 9]. However, anchoring these biomarkers to subtle cognitive and functional decline often relies on insensitive measures [10–12]. Moreover, the implementation of biomarker-based assessments for individuals at risk of AD faces criticism due to limited accessibility and invasiveness [1, 2, 13]. Consequently, there is a need for easily administered assessment measures correlating with brain changes in early AD stages to detect subtle impairments indicative of departure from healthy ageing [10, 14, 15].

In AD, a progressive decline in functional ability is observed across various domains of everyday life, including performing domestic chores, shopping for food and clothing and meal preparation [16]. Recognition is growing that functional impairment in MCI serves as a relevant predictor of progression to AD, challenging the traditional view of functional impairment as a downstream outcome of progressive neurodegenerative change [17–19]. Despite this, assessment tools to ascertain the presence of functional impairment often focus on basic daily tasks, potentially overlooking higher order functional changes such as managing finances [20–22]. Functional abilities hold differential sensitivity to the AD continuum, whereby more basic and higher order daily tasks are underpinned by different neural correlates, which are impacted by the disease at different stages of progression [23]. Therefore, more advanced and complex activities in daily life may hold greater potential to detect early impacts of cognition on functional abilities. This has led to calls for the development of functional assessment tools incorporating more complex activities of daily life susceptible to early manifestations of the AD prodrome [24–28].

Activities of daily living are typically categorised into basic ADLs (BADLs), instrumental ADLs (IADLs) and advanced ADLs (AADLs). BADLs refer to fundamental self-care tasks such as bathing, dressing and feeding, while IADLs encompass more complex activities required for independent living, including managing finances, shopping and meal preparation [29]. AADLs involve tasks that support societal roles and personal fulfilment, such as employment duties, volunteering or complex leisure activities [30]. Current functional assessment instruments, such as the Lawton and Brody IADL Scale (1969), the Disability Assessment for Dementia (DAD) [31] and the Amsterdam IADL Questionnaire (A-IADL-Q) [32], primarily focus on BADLs and IADLs. However, these tools typically lack integration of cognitive underpinnings such as memory binding, which may reduce their sensitivity for detecting early functional changes in prodromal AD.

Assessment tools incorporating instrumental and advanced activities of everyday life have demonstrated sensitivity to MCI and the early stages of AD [22, 28, 30, 33]. These tools encompass impairment in high-order functional domains, such as financial management, food preparation, medication management and the use of technology [34–37]. However, current measures that include these more complex tasks of everyday life often lack adequate psychometric properties, particularly content validity [38–41]. Additionally, scores from a significant proportion of available functional scales do not correlate with neuropsychological functions often assessed as part of the same batteries or protocols [42]. This may be suggestive of this identified lack of content validity within existing functional assessment tools, potentially indicating limited theoretical understanding of the constructs underpinning such scales. Given this, it has been argued that better measures are required, informed by recent advancements in experimental and biomarker research [28, 43–45]. One promising avenue involves designing measures based on cognitive markers derived from memory binding, which have shown sensitivity to early-stage AD [46] and hold potential towards ecological validity. This is relevant, as Slachevsky et al. [42] recently highlighted the relevance of meaningfulness in the assessment of functional decline, emphasising the need for person-centred approaches to functional assessment.

In cognition, memory binding refers to the mechanisms responsible for integrating or associating different pieces of information in short- and long-term memory [47]. Relational binding, holding associations between items and context or distinct items, is impaired in AD and prodromal MCI stages [46, 48–52]. This form of binding is suggested to rely on hippocampal activation, which becomes compromised in early-stage AD [53, 54]. Therefore, tasks dependent on this form of binding are recommended for early disease detection [46, 55]. Another form of binding, conjunctive binding, involves the integration of different representations of features within a unified object [56]. This form of binding has demonstrated impairment specific to MCI and the early stages of AD [50, 51, 57–59] while remaining preserved across normal ageing, non-AD dementias and depression [60–68], suggesting its potential as a sensitive cognitive marker for early AD. However, current assessments of these forms of binding often rely on laboratory-based tasks that are removed from everyday life [69]. Developing novel measures that assess binding in a more ecologically valid way, such as the ability to bind information required to perform complex everyday functions, may enhance diagnostic accuracy relative to consensus methods [70]. For example, while conjunctive binding functions support the ability to temporarily hold information on an object's identity, relational binding functions allow us to remember contextual information related to such identities (e.g., where the object was, alongside spatial arrangements). These are fundamental mnemonic processes subserving the normal function of working and episodic memory [71].

The Details of Function of Everyday Life (DoFEL) scale [70, 72] aims to refine functional assessment in early-stage AD by incorporating contemporary insights from memory binding. It evaluates functional ability while also assessing the capacity to bind information necessary for such functions as latent traits. This innovative approach holds potential for enhancing functional assessment compared to traditional methods [70, 73, 74]. The DoFEL assesses functional ability across 84 items constructed from complex activities of everyday life hypothesised to relate to conjunctive or relational binding. Traditional functional scales often measure observable tasks without capturing the underlying cognitive mechanisms, which may limit their sensitivity to early subtle impairments [42]. By explicitly incorporating memory binding, the DoFEL aims to detect functional difficulties that reflect prodromal AD-related cognitive changes before overt decline is apparent. The present study aimed to conduct a preliminary evaluation of the psychometric properties of the DoFEL, structured across three analytical steps.

  Step 1 (Exploratory Factor Analysis): To examine the latent factor structure of the DoFEL within a nonclinical sample and revise the scale by identifying items that best represent its underlying constructs.
  Step 2 (Content Validity Assessment): To evaluate the content validity of the revised DoFEL items by gathering expert dementia professionals' ratings of each item's relevance to memory binding and early functional decline in AD.
  Step 3 (Construct Validity Assessment): To assess the construct validity of the revised DoFEL by: (i) examining its correlation with established cognitive assessments (ACE-R and VSTMBT) and (ii) evaluating its discriminatory power to differentiate individuals with MCI from healthy older adults.

## 2. Methods

### 2.1. Design

This research utilised a three-step design. In Step 1, we investigated the latent factor structure of the DoFEL and revised the scale using data from a nonclinical sample. Step 2 involved administering a questionnaire to dementia professionals to assess the content validity of revised DoFEL items. For Step 3, we evaluated the construct validity of the revised DoFEL to differentiate between healthy ageing and MCI. Additionally, confirmatory factor analysis (CFA) was employed to assess the stability of the model in a separate sample.

### 2.2. Participants and Recruitment

In Step 1, a nonclinical sample comprising 559 healthy community-dwelling adults aged over 55 completed the DoFEL. Participants were recruited with the assistance of charitable organisations involved in supporting older adults in the UK. Recruitment was conducted between January and July 2023 through the online survey platform Qualtrics (Qualtrics, Provo, UT). Inclusion criteria required participants to be (i) aged 55 years or older, (ii) living independently in the community and (iii) self-reporting no history of neurological or psychiatric conditions. No additional exclusion criteria were applied at this stage. Demographic information for this sample is presented in Supporting Table 1.

For Step 2, eight dementia professionals with expertise in functional assessment for dementia and memory binding were approached. These professionals were purposively sampled based on their expertise in dementia assessment, functional evaluation and/or memory binding research. They were identified through professional networks of the research team, including colleagues from European universities and public health services (e.g., NHS memory clinics), ensuring a mix of academic and clinical perspectives. The sample included clinical neuropsychologists, geriatric neuropsychiatrists, occupational therapists and academic dementia researchers, all with more than 5 years of experience assessing cognitive and functional decline in AD. A closed questionnaire employing a Likert scale was used to gauge the relevance of revised DoFEL items to the construct of memory binding, alongside assessing the overall utility of the scale for detecting early functional changes associated with AD. Dementia professionals also had the option to provide anonymised feedback through free-text boxes. The number of eight professionals was chosen pragmatically, based on recommendations for content validity studies to include between five and 10 experts to provide sufficient breadth of feedback while ensuring the feasibility of analysis [75].

In Step 3, participants were recruited from a Scottish cohort study [56] comprising healthy older adults and individuals with MCI. Participants diagnosed with MCI were invited to attend the University of Edinburgh from NHS Lothian memory clinics. Simultaneously, healthy older adults were recruited from the Volunteer Panel for the School of Philosophy, Psychology and Language Sciences at the University of Edinburgh. Initial assessments were conducted at the university, during which demographic, health and neuropsychological data were collected. Inclusion criteria stipulated participants to be (i) aged 65 years or older and (ii) clinically diagnosed with MCI according to the gold-standard criteria established by Petersen [6, 76]. Within the MCI group, both amnestic and nonamnestic subtypes were included, as classification was based on Petersen's broad criteria rather than restricting to memory impairment subtypes alone. Alternatively, healthy older adults were confirmed through intact cognitive performance on the Addenbrooke's Cognitive Examination-Revised (score ≥ 88) at the time of testing, (iii) not diagnosed with any form of dementia and (iv) self-reporting no active psychiatric and neurological conditions.

All subjects provided informed consent in accordance with the Declaration of Helsinki. The procedures of this study were approved by the University of Edinburgh, School of Health in Social Science Research Ethics Committee (CLIN840).

### 2.3. Measures

#### 2.3.1. Demographic Characteristics

Sociodemographic data, including age, years of education and sex, were collected to discern the characteristics of the sample under investigation. Participants were also asked to disclose any active neurological or psychiatric conditions.

#### 2.3.2. DoFEL

The DoFEL was developed to operationalise functional activities underpinned by cognitive binding processes implicated in prodromal AD stages [77]. Item generation involved an iterative, theory-driven process, whereby daily activities were identified based on prior experimental paradigms assessing conjunctive and relational binding [50, 78] and then translated into ecologically valid functional tasks through expert discussion. This process included reviewing existing functional scales to identify relevant domains and constructing items hypothesised to depend specifically on either conjunctive (e.g., integrating object features) or relational binding (e.g., associating objects with spatial or contextual information).

The DoFEL scale assesses 84 items of functional ability across several domains of everyday life. Each item has been conceptualised to depend on either conjunctive or relational binding forms. For instance, ‘Difficulty recognising traffic signs' has been categorised as depending on conjunctive binding, whereas ‘Difficulty using landmarks for directions when driving' has been classified as a function of relational binding. The DoFEL contains 53 items hypothesised to depend on relational binding and 31 items posited to be dependent on conjunctive binding. A full breakdown of items by binding type is provided in Supporting Table 10. A copy of the original DoFEL scale is provided in Table A1 in the appendix.

A Likert scale is utilised from zero to three (0 = ‘not applicable', 1 = ‘never', 2 = ‘occasionally' and 3 = ‘frequently'), which attempts to capture the degree of functional impairment within an individual's daily life. For each participant, a standardised DoFEL score (between 0 and 1) and standardised total subdomain scores (each between 0 and 1) are obtained by dividing the total score (sum of responses to items answered) by the maximum score (where the maximum score refers to the number of items answered multiplied by 3). Items with a response of 0 (‘not applicable') are excluded from calculations.

Given that the DoFEL is a novel assessment, its psychometric properties remain under investigation. However, a higher score reflects greater difficulty in activities of daily living. As Slachevsky et al. [42] highlighted, a gap within available functional scales is how they approach the ‘what' question. For example, the Lawton and Brody [29] IADL scale asks whether they can operate the telephone, granting them a score of 0 if they respond, ‘Does not use the telephone at all'. Operating a telephone is an instrumental ability that relies on perceptual, mnemonic, executive and motor functions. For instance, patients can receive a score of 1 on this item of the scale even if they fail to associate a number with a person (e.g., if they ‘Dial a few well-known numbers', they receive 1). By exploring the constituent parts of such complex abilities and their cognitive correlates, we stand a better chance to detect early subtle functional deficits, which can signal risk for progression to dementia.

Considering the above evidence, in the present study, we hypothesised that DoFEL items within each functional domain should be associated with first-order factors of conjunctive and relational binding. These items have been developed based on specific recognition tasks grounded in binding theory [72]. However, to ensure ecological validity, these tasks were incorporated into a measure of functional activity, resulting in conceptually similar items across multiple functional domains [74]. This suggests a hierarchical structure, with binding types serving as first-order factors summarising item-level data from subscales and a higher order factor of functional activity summarising the common information related to each subscale.

#### 2.3.3. Addenbrooke's Cognitive Examination-Revised

The ACE-R [79] is the revised version of the original Addenbrooke's Cognitive Examination [80], a widely used assessment to detect cognitive change and differentiate dementia subtypes. This revised version of the ACE consists of 26 tasks across the five broad cognitive domains of Attention and Orientation, Memory, Visuospatial Function, Language and Verbal Fluency.

A trained individual typically administers the ACE-R over 20 min, and a general score is computed out of 100 on completion. Lower scores reflect reduced cognitive ability, with scores of 88 and below indicating cognitive impairment [79]. The ACE-R has demonstrated high diagnostic accuracy in the detection of MCI [81, 82] and has been shown to have high reliability within the measurement of early cognitive change, with Cronbach alpha coefficients greater than 0.8 reported [83].

#### 2.3.4. Visual Short-Term Memory Binding Task

The VSTMBT [50, 84] is an experimental assessment designed to evaluate cognitive performance, specifically focussed on memory-binding abilities. The test consists of three conditions using visual arrays of two or three stimuli; for a comprehensive description of the task's psychophysics properties, refer to Parra et al. [50]. Briefly, the VSTMBT presents visual arrays of two or three abstract shapes displayed in random positions of a ‘3 × 3' virtual grid. After an initial fixation cross (1000 ms), a study display was presented for 2000 ms, followed by a 900-ms unfilled retention interval. The test display was then presented and remained on until participants responded. Participants were asked to detect whether a change occurred between the study and the test display or if the stimuli remained the same. The task consisted of two conditions; the Shape Only condition assessed VSTMBT for single features. The Binding condition assessed VSTMBT for shape-colour bindings. During the Shape Only condition, participants were presented with either two or three black shapes, and in the Binding condition, two or three shapes were presented, each in a different colour. To be able to detect such changes, participants had to remember either the individual shapes or the combinations of shape and colour (i.e., Binding condition) presented in the study display. Each condition consisted of 32 trials, of which 50% presented arrays of two stimuli and the remaining 50% presented three stimuli. Scoring for the VSTMBT is based on the percentage of correct recognition. Therefore, a higher percentage indicates less impairment in memory-binding ability. In this study, we specifically investigated the performance of the ‘Shape-Colour Binding' condition within this task, as it has demonstrated the highest classification power in AD studies [56, 63, 85].

### 2.4. Data Analysis

#### 2.4.1. Exploratory Factor Analysis (Step 1)

Principal axis factoring (PAF) with oblimin rotation was performed to explore latent constructs within the DoFEL subscales. Candidate items for removal were considered, and postrotation factors were extracted for each DoFEL subscale. The number of factors retained was determined through parallel analysis [86], and DoFEL items with communalities (h^2^) scores greater than 0.35 were retained [87]. Eigenvalues were examined as part of this process, with factors retained if their real-data eigenvalues exceeded those generated through parallel analysis, indicating they explained more variance than expected by chance. Multicollinearity between DoFEL items, Bartlett's test of sphericity and the Kaiser–Meyer–Olkin measure of sampling adequacy were examined to ensure that the assumptions for conducting PAF were met.

#### 2.4.2. Content Validity Assessment (Step 2)

The Average Content Validity Index (Ave-CVI) [88] was used to assess the level of agreement among dementia professionals regarding the relevance of DoFEL items to the construct of binding. Qualitative responses were also reviewed and discussed by the research team to evaluate the acceptability of scale items.

#### 2.4.3. CFA

CFA was employed to assess the stability of the dual-factor model of conjunctive and relational binding across the revised DoFEL subscales. The suitability for CFA analysis was evaluated through Bartlett's test of sphericity, examination of the anti-image covariance matrix and the Kaiser–Meyer–Olkin measure of sampling adequacy [89]. The latent variable of binding was scaled by fixing the variance to 1.00 [90]. Model goodness of fit was evaluated using a range of fit indices following guidelines by Kline [91] and Brown [92]. This guidance stipulated that an acceptable model fit should have a root-mean-square error of approximation (RMSEA) close to 0.06 or less, a comparative fit index (CFI) greater than roughly 0.90, standardised root-mean-square residuals (SRMR) close to 0.08 or less and a Tucker–Lewis index (TLI) close to 0.90 or greater. Additionally, the model chi-squared (*χ*^2^) statistic was assessed to evaluate overall fit. However, this was interpreted cautiously due to its sensitivity to sample size, with higher *p*-values suggesting a better model fit [93].

#### 2.4.4. Construct Validity Assessment (Step 3)

Construct validity was assessed by examining the relationship between functional performance on the revised DoFEL and cognitive performance on the ACE-R and VSTMBT. Additionally, binomial logistic regression was employed to evaluate the sensitivity of revised DoFEL total scores in predicting MCI classification compared to healthy controls. Receiver operating characteristic (ROC) curve analysis was used to assess the discriminatory ability of the revised DoFEL by plotting sensitivity against 1-specificity at various thresholds, with the area under the curve (AUC) indicating overall diagnostic accuracy (values closer to 1.0 indicate excellent discrimination, while values near 0.5 indicate chance performance) [94]. AUC values were also used for the comparison of logistic regression models for revised DoFEL total scores and the original DoFEL total scores in distinguishing between MCI and healthy ageing.

## 3. Results

### 3.1. Exploratory Factor Analysis (Step 1)

#### 3.1.1. Sample Demographics

The nonclinical sample contained 559 responses to the DoFEL from cognitively intact participants without psychiatric or neurological illness. Participants were aged between 55 and 94 and included 253 male participants, 304 female participants, and two participants who preferred not to disclose their sex. There was a wide range within participant education level, with the average years of education for the sample being 15.8 years. Further details on the demographics of this sample are presented in the Supporting Table 1.

#### 3.1.2. Factorial Validity of DoFEL Subscales

PAF was used to explore the first-order factor structure of item-level data from DoFEL subscales. Preliminary assumption testing indicated that data from DoFEL subscales were likely factorisable, as demonstrated in the Supporting Table 2.

The exploratory factor analysis results for the Shopping and Money subscale demonstrated a two-factor solution, explaining 62.8% and 14.2% of the variance. Three items were removed from this subscale due to communalities falling below the 0.35 threshold. Similarly, the Objects and People subscale showed a two-factor solution (33.2% and 14.6% variance explained), with four items removed based on the same communality criterion. The Technology and Communication subscale also showed a two-factor solution (62.1% and 10.6%), and 13 items were removed. In all of these subscales, the identified factors appeared indicative of either relational binding or conjunctive binding.

The Transport and Mobility subscale demonstrated a single-factor solution, accounting for 78.4% of the variance, with one item removed. Loading patterns revealed that conjunctive and relational binding appeared to load negatively and positively, respectively, on the identified factor. The Domestic Chores subscale exhibited a two-factor solution, explaining 46.0% and 34.2% of the variance, with five items removed. Similarly, the Work and Social Life and Health and Lifestyle subscales showed two-factor solutions, explaining 58.4% and 20.9%, and 60.5% and 19.7% of the variance, respectively. These factors within the subscales were also indicative of either conjunctive or relational binding. In the Work and Social Life subscale, five items were removed, while in the Health and Lifestyle subscale, nine items were removed. Items were removed from all subscales if their communality scores were below the recommended cut-off of 0.35 [87].

The examination of factor loadings and communalities across the various subscales of the DoFEL revealed consistent patterns indicative of relational and conjunctive binding. Items associated with relational binding displayed high factor loadings and communalities, suggesting their robust contribution to this construct as a latent trait being measured. Additionally, conjunctive binding items demonstrated notable factor loadings and communalities, emphasising their role as a latent trait of the functional tasks included in the DoFEL. This analysis suggests a potential connection between these binding processes and functional task performance on the DoFEL. Specifically, it hints at how an individual's ability to bind information may influence performance in various functional domains. However, further confirmatory analyses and complementary methodologies are required to understand these observed relationships.

The outcomes of the exploratory factor analysis are detailed in Table 1, while Supporting Tables 3, 4, 5, 6, 7, 8, 9 provide the factor loadings and communalities for each respective subscale.

### 3.2. Content Validity of the Revised DoFEL (Step 2)

Eight dementia professionals provided feedback on the revised DoFEL items in regard to the overall item utility and the relevance of items to the concept of binding. None of the dementia professionals suggested any item be excluded from the scale, and all commented that items appeared useful for assessing early changes to cognition indicative of AD dementia.

Overall, the proportion of agreement between professionals for the relevance of items dependent on relational binding was 71.9% and 60.6% for those classified as dependent on conjunctive binding. The Ave-CVI was used to determine the consensus among professionals regarding the relevance of the various items to either conjunctive or relational binding. The Ave-CVI was 0.44 for relational items and 0.21 for those classified as conjunctive. Overall, this indicates poor content validity for the scale's relevance to binding, as reflected by Ave-CVI values falling below the acceptable threshold of 0.8, as suggested by Almanasreh et al. [95]. Further details of the ratings given for each item by dementia professionals are outlined in Supporting Table 10.

The dementia professionals commented that functional tasks with a broader description were more likely to encompass specific functional components that could depend on either form of binding or other cognitive functions, such as executive functioning. It was outlined that this limited confidence in attributing task dependency to either form of binding despite maintaining that those items appeared useful for the assessment of early cognitive change in AD dementia and MCI.

Overall, the revised DoFEL included 44 items. This included 20 hypothesised to depend on conjunctive binding and 24 considered to rely on relational binding. We have included a copy of the revised DoFEL as an appendix (see Table A2).

### 3.3. CFA of the Revised DoFEL

#### 3.3.1. Sample Demographics

This sample comprised 135 participants (60.7% female) recruited as healthy controls for the aforementioned Scottish cohort study. The sample ranged in age from 65 to 93, with a mean age of 73.9 years (SD = 7.4). In general, years of education for participants in this sample were high, with the average years of education being 14.2 years.

#### 3.3.2. CFA

CFA was conducted on the items of each subscale within the revised DoFEL. There were no missing or erroneous values within the dataset, and prior assumption testing to ascertain the appropriateness of employing CFA within this dataset suggested the data were likely factorisable. Details of the assumption testing are presented in Supporting Table 11.

Evaluating the model fit across various subscales of the DoFEL, the Shopping and Money subscale demonstrated an overall acceptable fit, with the exception of the RMSEA index. Conversely, the two-factor model for the Objects and People subscale did not attain an acceptable fit in this sample, with slight deviations in fit indices. The Technology and Communication subscale exhibited a well-fitting two-factor model, while the Transport and Mobility subscale model demonstrated an acceptable fit, except for the RMSEA. Moreover, the two-factor model for the Domestic Chores subscale showed a satisfactory fit. For the Work and Social Life subscale, the dual-factor solution displayed a nearly acceptable fit, albeit with minor discrepancies in RMSEA and TFI Indices. The Health and Lifestyle subscale two-factor model did not achieve an acceptable fit. Detailed fit statistics for each subscale are provided in Table 2.

### 3.4. Construct Validity of the Revised DoFEL (Step 3)

#### 3.4.1. Sample Demographics

This sample comprised 49 participants diagnosed with MCI and 33 healthy controls recruited for the aforementioned Scottish cohort study. There was a diverse range of ages and educational attainment levels across the total sample and diagnostic subgroups, as shown in Table 3.

A large body of literature has demonstrated that the effects of age and level of education can significantly influence performance on assessments of function and cognitive ability [96, 97]. Therefore, a Mann–Whitney U test was run to determine if the demographic characteristics of age and years of education were matched between samples, as the assumption of normality per Shapiro–Wilk's test was not met [98].

There was no statistically significant difference in age between the MCI group (*Mdn* = 73) and healthy controls (*Mdn* = 73), *U* = 738, *z* = 2.88, and *p*=0.51. However, a statistically significant difference was observed in educational attainment between the MCI group (*Mdn* = 16) and healthy controls (*Mdn* = 0.12), *U* = 459, *z* = 1.85, and *p*=0.001.

Data informing on overall neuropsychological test performance for both MCI and the healthy control group have been included in Supporting Table 12.

#### 3.4.2. Correlation Between Revised DoFEL and ACE-R

Pearson's product-moment correlation was run to assess the relationship between the revised DoFEL and ACE-R scores in a sample of people across healthy ageing to MCI. Preliminary analyses showed the relationship to be linear, with both variables normally distributed and no significant outliers.

There was a statistically significant, strong negative correlation between the total scores of the revised DoFEL scale and the ACE-R, *r*(82) = −0.66, *p* < 0.001, with revised DoFEL scores explaining 43.95% of the variation in ACE-R scores. This suggests that as performance on the DoFEL decreases (higher DoFEL score), the performance on the ACE-R decreases.

#### 3.4.3. Correlation Between Revised DoFEL and the VSTMBT

Pearson's product-moment correlation was run to assess the relationship between the revised DoFEL scores and performance on the VSTMBT. Preliminary analyses showed the relationship to be linear, with both variables normally distributed and no significant outliers.

There was a statistically significant, moderate negative correlation between the total scores of the revised DoFEL scale and the VSTMBT, *r*(82) = −0.52, and *p*=0.003, with revised DoFEL scores explaining 26.83% of the variation in VSTMBT scores. This suggests that as performance on the DoFEL decreases (higher DoFEL score), the performance on the memory-binding task also decreases.

#### 3.4.4. Predicting MCI: Binomial Logistic Regression Analysis of Revised DoFEL Scores

Binomial logistic regression was performed to ascertain the influence of the total revised DoFEL scores on the likelihood that participants have MCI. Following the meeting of appropriate assumptions, the logistic regression model was statistically significant, *χ*^2^ (1) = 75.5, *p* < 0.001, and *R*^2^ = 0.81. The model for the revised DoFEL scores explained 81.3% of the variance in predicting MCI, and the area under the ROC curve was 0.95, which is an excellent level of discrimination, according to Homser et al. [94].

Considering the original DoFEL scores, this logistic regression model was statistically significant. The original DoFEL model explained 13.8% of the variance in predicting MCI, *χ*^2^ (1) = 6.90, *p*=0.02, and *R*^2^ = 0.14, with an AUC value of 0.66.

## 4. Discussion

### 4.1. Summary of Findings

The main purpose of this research was to investigate the structure, content and construct validity of the DoFEL. Specifically, the study aimed to (i) examine the latent factor structure of the DoFEL and revise the scale by identifying items representing conjunctive and relational memory binding (Step 1), (ii) evaluate the content validity of revised DoFEL items by gathering expert ratings on their relevance to memory binding and early functional decline in AD (Step 2) and (iii) assess the construct validity of the revised DoFEL by examining its correlation with established cognitive assessments (ACE-R and VSTMBT) and its ability to differentiate individuals with MCI from healthy older adults (Step 3). An additional aim was to investigate the representation of memory binding within a measure of functional activity, contributing to the literature on the potential integration of cognitive markers to enhance functional assessment in prodromal AD stages.

This study had five key findings: (1) The constructs of conjunctive and relational binding appeared to be represented as dimensions of items included in the DoFEL subscales. However, the factor structure across all subscales was not replicated in a differing sample, suggesting further scale revision would be of benefit. (2) Dementia professionals considered the DoFEL a useful tool for detecting functional impairment in early-stage AD. However, the relevance of certain functional tasks to binding was questionable due to their broad nature. (3) Functional performance measured by the revised DoFEL strongly correlated with cognitive performance assessed by the ACE-R. (4) Performance on the DoFEL also showed a moderate correlation with performance on an experimental measure of memory binding. (5) The revised DoFEL could differentiate those on a healthy ageing trajectory from individuals diagnosed with MCI, demonstrating comparability with the ACE-R.

Regarding the structural (construct) validity of the DoFEL, the results of the factorial analysis support a two-factor model of conjunctive and relational binding as latent traits of functional performance measured by the DoFEL. There was evidence for the stability of the model across samples in the Shopping and Money, Technology and Communication, Transport and Mobility and Domestic Chores subscales. However, the model fit was less than optimal in the Objects and People, Work and Social Life and Health and Lifestyle subscales. This variability in model fit suggests that while the two-factor model captures specific dimensions of functional performance, further refinement is required to capture the complexity of functional performance in these domains. The extent to which functional performance in daily living tasks is supported by memory binding remains underexplored [46, 57, 99]. However, there remains an interesting theoretical relationship between memory binding as an underlying trait of functional performance in the DoFEL. Overall, this may imply that formulating questions based on specific cognitive markers may have the potential to unveil subtle functional impairments that often elude detection in prodromal AD stages.

Considering the content validity of the revised DoFEL post-PAF, the results reveal that all functional tasks included were considered useful for assessing early changes to cognition indicative of AD dementia. However, the level of agreement in attributing performance on these tasks to the construct of memory binding was poor. The finding that dementia professionals identified the items included within the revised DoFEL as useful for evaluating functional activity within prodromal AD stages corroborates the growing body of evidence demonstrating impairment in more complex activities of daily living in the early stages of AD [17]. However, the overall poor agreement between professionals on whether revised DoFEL items appeared relevant to conjunctive or relational binding suggests that further item modification is required to integrate the concept of memory binding into a measure of functional ability. It also indicates a need for further research to determine the extent to which other cognitive processes may contribute to DoFEL task performance in addition to memory binding. For example, considering recent recommendations by [42], future efforts should involve patients and caregivers to explore which of these items they consider to be more meaningful or which are missing [100].

The final aim of this research was to examine the construct validity of the revised DoFEL. To this end, significant negative correlations were observed between functional performance as measured by the revised DoFEL and cognitive performance on the ACE-R and VSTMBT. This suggests that cognitive performance decreased (lower ACE-R and VSTMBT scores) as functional performance decreased (higher revised DoFEL scores). While this relationship is not deterministic, it is consistent with research documenting that people with higher levels of cognitive dysfunction tend to have more limitations in higher level activities of daily living [101]. While recently developed scales to assess difficulties in everyday functioning in dementia have shown excellent discrimination between noncognitive impairment and mild dementia, such as the A-IADL-Q, further research is required to determine which functional tasks effectively map onto cognitive performance and may meaningfully differentiate healthy ageing from mild cognitive decline [42, 102, 103]. This appears to be a key strength of the revised DoFEL, whereby the functional tasks included demonstrate strong construct validity with the cognitive underpinnings known to be impacted in preclinical AD stages. Moreover, the binomial regression analysis demonstrated that performance on the revised DoFEL effectively differentiated individuals with MCI and healthy controls. These results are consistent with research, demonstrating that measures incorporating complex functional tasks can detect cognitive decline in prodromal AD stages [101, 104, 105]. This is important as it indicates that the DoFEL might capture functional decline specific to early-stage AD and also predict an individual's cognitive abilities before undergoing extensive testing.

Recent instruments, for example, the Activities of Daily Living Inventory (ADLI; [106]), provide comprehensive assessments of daily functioning across multiple domains, supporting the characterisation of functional abilities in older adults. However, unlike existing measures, the DoFEL was conceptually designed to integrate specific cognitive processes, namely conjunctive and relational memory binding, theorised to underlie functional performance and aspects of cognitive impairment in prodromal AD stages. This theoretically grounded approach may enhance its sensitivity to subtle functional decline before overt impairment is observable, addressing an important gap in current functional assessment tools. Overall, the present study provides preliminary evidence that integrating cognitive theory into functional assessment is a promising direction for improving early detection of AD and offers a foundation for future refinement and validation of the DoFEL as an ecologically valid, cognitively informed tool.

### 4.2. Limitations and Future Directions

Taken together, this study has attained preliminary information supporting conjunctive and relational binding as dimensions of functional activity in the DoFEL. It has also shown the ability of the revised DoFEL to discriminate MCI from healthy ageing regarding overall functional performance. However, despite these promising findings, several limitations require discussion. The main limitation is that while this study has investigated the DoFEL regarding structural, content and construct validity, further information regarding the reliability and sensitivity over time of the DoFEL is required to conclude the recommendation for implementing this assessment in both research and clinical practice.

Due to the nature of participant data within the cohort study, it was not possible to compare the revised DoFEL against gold-standard cognitive and functional assessments. Therefore, further research is required to determine whether the DoFEL is superior or equivalent to existing methods of diagnosis. The DoFEL is a unique tool assessing function and memory binding within one measure. However, further research is required to determine the translational potential of memory binding within a functional assessment tool. This study suggests that functional assessment tools based on the specific cognitive vulnerabilities caused by AD are a promising way to improve early detection. However, it is important to ascertain the benefits of this approach over consensus diagnostic methods.

A further limitation of this research is the reliance on ACE-R scores to define healthy ageing. To be included as healthy older adults, participants needed to attain a score above 88 on the ACE-R, as scores below this threshold typically indicate cognitive impairment [79]. While the ACE-R assessment alone is helpful for identifying individuals with MCI [107], it was not designed to provide a definitive diagnosis of AD [6, 108]. Therefore, without the use of consensus diagnostic approaches or data from accompanying tests, such as neuropsychological and neuroimaging assessments, it is plausible that some participants classified as healthy may have had cognitive impairment indicative of MCI. Future studies incorporating more comprehensive testing would offer additional validation for healthy ageing and ensure greater accuracy in comparison.

An additional limitation of this research is related to the exclusion criterion. In studies assessing a new assessment to support the detection of MCI or early-stage AD, it is common to exclude individuals with a diagnosis of depression using a screening tool, as depression can impact cognitive performance [109]. However, due to the availability of data, it was not possible to exclude individuals based on this factor. Therefore, while individuals did not disclose active psychiatric conditions, there may be individuals included with symptoms of depression that may influence performance outcomes.

Another limitation is that the revised DoFEL scores were calculated using data derived solely from item responses of the original scale, thereby excluding items not retained in the revised version. This omission could potentially affect the generalisability of the revised DoFEL scores, as they lack direct validation through administering the scale to new participants. While the DoFEL scoring format may differ from some functional assessments in that higher scores reflect greater difficulty, this structure aligns with many clinical tools in which elevated scores denote increased symptom severity.

Additionally, further research in larger and more diverse populations would strengthen the findings of this work. It is recognised that MCI and AD are conditions that impact people across the globe, and currently, there is a lack of tools available for use in developing nations [110]. Therefore, the DoFEL must be evaluated in a range of different cultural contexts, so it is applicable as an assessment for a range of populations. Such studies would indeed be important, as memory binding does not appear to be influenced by the differential effects of culture, education level and socio-economic status [111, 112]. However, in the present study, a significant difference in educational attainment was observed between the clinical and nonclinical groups, so future research is needed to control for this variable and assess its potential impact on DoFEL performance.

Furthermore, while the DoFEL was developed to enhance early detection of AD by targeting cognitive vulnerabilities such as memory binding deficits, particularly conjunctive binding, which has demonstrated AD-specific impairment, its generalisability to other dementia subtypes is likely limited. Given that different dementias present with distinct cognitive profiles and memory binding impairments have not been consistently observed outside AD, future research may focus on developing functionally grounded assessments targeting cognitive vulnerabilities characteristic of other dementia types, rather than directly adapting the DoFEL.

### 4.3. Conclusion

In summary, this research provides initial evidence that the DoFEL is a promising measure of functional activity, capable of discriminating MCI from healthy ageing. This offers a potential avenue for the early detection of AD by identifying subtle functional changes in individuals with MCI, including both amnestic and nonamnestic subtypes, through ecologically valid approaches using cognitive markers to enhance functional assessment within the early stages of AD. However, ongoing refinement is needed to establish the reliability and broader applicability of the DoFEL in both research and clinical practice.

## Figures and Tables

**Table 1:** Results of the principal axis factoring.

Subscale	Number of factors	Variance explained (%)	Real eigenvalues	Eigenvalues generated through parallel analysis	Items removed
Shopping and Money	2	62.8	5.02	1.18	3
		14.2	1.14	1.12	

Objects and People	2	33.2	4.31	1.26	4
		14.6	1.89	1.19	

Technology and Communication	2	62.1	6.96	1.33	13
		10.6	1.10	1.07	

Transport and Mobility	1	78.4	5.49	1.16	1

Domestic Chores	2	46.0	1.84	1.19	5
		34.2	1.37	1.13	

Work and Social Life	2	58.4	4.09	1.23	5
		20.9	1.47	1.16	

Health and Lifestyle	2	60.5	3.63	1.27	9
		19.7	1.18	1.14	

*Note:* The table presents an analysis of the DoFEL subscales, detailing the number of factors identified, the variance explained by each factor, the real eigenvalues obtained and the corresponding eigenvalues generated through parallel analysis.

**Table 2:** CFA results summary for the DoFEL subscales.

Subscale	*X* ^2^	RMSEA	CFI	SRMR	TLI
Shopping and Money	8.89^∗^	0.115	0.955	0.049	0.943
Objects and People	85.1^∗∗^	0.163	0.839	0.108	0.740
Technology and Communication	6.92^∗^	0.073	0.984	0.032	0.961
Transport and Mobility	8.48^∗^	0.155	0.977	0.039	0.930
Domestic Chores	1.16^∗^	0.034	0.994	0.025	0.994
Work and Social Life	7.30^∗∗^	0.216	0.950	0.052	0.701
Health and Lifestyle	79.9^∗∗^	0.333	0.878	0.072	0.856

*Note:X* ^2^ = chi-squared statistic, SRMR = standardised root mean square.

Abbreviations: CFI = comparative fit index, RMSEA = root-mean-square error of approximation, TLI = Tucker–Lewis index.

^∗^ *p* < 0.05.

^∗∗^ *p* < 0.001.

**Table 3:** Demographic characteristics of the sample by diagnostic group.

Characteristic	Subgroups of the sample		
	Total sample	MCI	HOA
Mean age (years)	73.8	74.5	73.4
Mean educational attainment level (years)	14.5	15.7	12.8
Sex (% female)	62.2%	75.5%	42.4%

**Table 4:** Details of functions of everyday life (DoFEL).

			**Not applicable ⓪—never ①—occasionally ②—frequently ③**	
**Shopping and Money**				

1	Misplaces money or forgets where to find it		⓪—①—②—③	R
2	Difficulty recognising coins or notes (bills)		⓪—①—②—③	C
3	Difficulty handling money when paying or receiving change		⓪—①—②—③	R
4	Difficulty recognising common products in supermarkets or shops		⓪—①—②—③	C
5	Forgets the name of products commonly shopped for		⓪—①—②—③	R
6	Forgets the location of products at home or in supermarkets		⓪—①—②—③	R
7	Difficulty remembering the way to the supermarket or back home		⓪—①—②—③	R
8	Difficulty remembering items without a shopping list		⓪—①—②—③	C
Total				

**Objects and People**				

9	Difficulty recognising personal items (keys, clothing)		⓪—①—②—③	C
10	Difficulty remembering the location of personal items		⓪—①—②—③	R
11	Difficulty recognising new items (a new home appliance)		⓪—①—②—③	C
12	Difficulty recognising familiar items (gardening tools, a book)		⓪—①—②—③	C
13	Difficulty recognising faces of well-known people		⓪—①—②—③	C
14	Difficulty recognising faces of people recently met		⓪—①—②—③	C
15	Difficulty recalling the name of well-known people		⓪—①—②—③	R
16	Difficulty recalling the name of people recently met		⓪—①—②—③	R
17	Forgets where friends and family members live		⓪—①—②—③	R
18	Difficulty recognising the house of old friends or family members		⓪—①—②—③	C
19	Difficulty recognising the house of new friends		⓪—①—②—③	C
20	Forgets where the car was parked		⓪—①—②—③	R
21	Difficulty recognising own car (confuses it with a similar car)		⓪—①—②—③	C
Total				

**Technology and Communication**				

22	Forgets well-known telephone numbers		⓪—①—②—③	C
23	Forgets recently learned telephone numbers		⓪—①—②—③	C
24	Difficulty dialling	Mobile phones	⓪—①—②—③	C
24a		Landline phones	⓪—①—②—③	C
25	Forgets where the landline phone is at home		⓪—①—②—③	R
26	Forgets where the mobile phone was left		⓪—①—②—③	R
27	Difficulty recognising own mobile phone		⓪—①—②—③	C
28	Difficulty recognising own mobile phone's ring tone		⓪—①—②—③	C
29	Difficulty recalling pin codes (such as in cash machines)		⓪—①—②—③	C
30	Difficulty recognising the bank card among other cards		⓪—①—②—③	C
31	Difficulty operating the cash machine		⓪—①—②—③	R
32	Difficulty recalling the pin code of different bank cards		⓪—①—②—③	R
33	Difficulty changing TV channels (operating remote controllers)		⓪—①—②—③	C
34	Forgets the channel of commonly watched TV programmes		⓪—①—②—③	R
35	Difficulty recalling the name of TV characters		⓪—①—②—③	R
36	Difficulty keeping track of storylines in TV programmes		⓪—①—②—③	R
37	Forgets the programmes where famous TV characters act or acted		⓪—①—②—③	R
38	Forgets the username and password of email accounts		⓪—①—②—③	C
39	Forgets the email account provider (Hotmail, Yahoo, Gmail)		⓪—①—②—③	C
40	Difficulty recognising or operating online communication	Emails	⓪—①—②—③	R
		Skype	⓪—①—②—③	R
41	Forgets how to use email (send a message, retrieve a message)		⓪—①—②—③	C
Total				

**Transport and Mobility**				

42	Forgets basic actions when driving (how to change lights)		⓪—①—②—③	R
43	Difficulty recognising traffic signs		⓪—①—②—③	C
44	Difficulty driving in familiar places		⓪—①—②—③	R
45	Difficulty driving in new places		⓪—①—②—③	R
46	Difficulty using landmarks for directions when driving		⓪—①—②—③	R
47	Difficulty using landmarks when walking to familiar places		⓪—①—②—③	R
48	Difficulty using landmarks when walking to unfamiliar places		⓪—①—②—③	R
Total				

**Domestic chores**				

49	Difficulty operating well-known/previously used electric appliances		⓪—①—②—③	C
50	Forgets the function of well-known/previously used electric appliances		⓪—①—②—③	R
51	Forgets the location of electric appliances at home		⓪—①—②—③	R
52	Forgets where to find ingredients when cooking		⓪—①—②—③	R
53	Difficulty selecting ingredients when cooking		⓪—①—②—③	R
54	Mixes up ingredients from different recipes		⓪—①—②—③	R
55	Use the wrong kitchen utensils when cooking		⓪—①—②—③	R
56	Forgets the name of ingredients when cooking		⓪—①—②—③	R
57	Difficulty adding the correct amount of ingredients to the food		⓪—①—②—③	R
Total				

**Work and Social Life**				

58	Forgets the name of people at work		⓪—①—②—③	R
59	Forgets the date and time of tasks at work		⓪—①—②—③	R
60	Difficulty remembering things that happened at work		⓪—①—②—③	R
61	Difficulty allocating priority to tasks and remembering priorities		⓪—①—②—③	R
62	Difficulty getting well dressed to go to work and social meetings		⓪—①—②—③	R
63	Forgets daily work routines		⓪—①—②—③	C
64	Difficulty recognising friends with whom interacts regularly		⓪—①—②—③	C
65	Forgets the name of new colleagues or friends		⓪—①—②—③	R
66	Forgets good manners during social interactions (thanking, greeting)		⓪—①—②—③	R
67	Shows occasional inappropriate behaviours (say or does rude things)		⓪—①—②—③	R
68	Difficulty distinguishing between social and work affairs		⓪—①—②—③	R
Total				

**Health and Lifestyle**				

69	Forgets to take medication in time		⓪—①—②—③	R
70	Forgets who prescribed the medication		⓪—①—②—③	R
71	Difficulty recognising the medication (what the red pill is for)		⓪—①—②—③	C
72	Forgets where medicines are kept		⓪—①—②—③	R
73	Difficulty recognising unhealthy food which was previously avoided		⓪—①—②—③	R
74	Difficulty recognising changes in own physical status (weight, energy)		⓪—①—②—③	C
75	Difficulty recognising healthy food which was previously preferred		⓪—①—②—③	R
76	Forgets what activities are healthy (exercise, reading, leisure)		⓪—①—②—③	R
77	Forgets where to practice healthy activities (gym, park)		⓪—①—②—③	R
78	Forgets with whom to practice healthy activities		⓪—①—②—③	R
79	Forgets regular past-time activities (walks, reading)		⓪—①—②—③	C
80	Withdrawn from habitual leisure		⓪—①—②—③	C
81	Forgets where to practice leisure activities (community centre)		⓪—①—②—③	R
82	Forgets how to practice leisure activities (play bingo, cards)		⓪—①—②—③	R
Total				
Grand total				

**Table 5:** Details of functions of everyday life-revised (DoFEL-R).

			**Not applicable ⓪—never ①—occasionally ②—frequently ③**	
**Shopping and Money**				

1	Misplaces money or forgets where to find it		⓪—①—②—③	R
2	Difficulty handling money when paying or receiving change		⓪—①—②—③	R
3	Difficulty recognising common products in supermarkets or shops		⓪—①—②—③	C
4	Forgets the location of products at home or in supermarkets		⓪—①—②—③	R
5	Difficulty remembering items without a shopping list		⓪—①—②—③	C
Total				

**Objects and People**				

6	Difficulty recognising personal items (e.g., keys, clothing)		⓪—①—②—③	C
7	Difficulty remembering the location of personal items		⓪—①—②—③	R
8	Difficulty recognising familiar items (e.g., gardening tools, a book)		⓪—①—②—③	C
9	Difficulty recognising faces of well-known people		⓪—①—②—③	C
10	Difficulty recalling the name of well-known people		⓪—①—②—③	R
11	Difficulty recalling the name of people recently met		⓪—①—②—③	R
12	Difficulty recognising the house of new friends		⓪—①—②—③	C
13	Forgets where the car was parked		⓪—①—②—③	R
14	Difficulty recognising own car (e.g., confuses it with a similar car)		⓪—①—②—③	C
Total				

**Technology and Communication**				

15	Forgets recently learned telephone numbers		⓪—①—②—③	C
16	Difficulty dialling	Mobile phones	⓪—①—②—③	C
16a		Landline phones	⓪—①—②—③	C
17	Difficulty recognising own mobile phone		⓪—①—②—③	C
18	Difficulty recognising own mobile phone's ring tone		⓪—①—②—③	C
19	Difficulty operating the cash machine		⓪—①—②—③	R
20	Difficulty recalling the pin code of bank cards		⓪—①—②—③	R
21	Difficulty keeping track of storylines in TV programmes		⓪—①—②—③	R
22	Difficulty recognising and/or operating communication by email		⓪—①—②—③	R
Total				

**Transport and Mobility**				

23	Forgets basic actions when driving (e.g., how to change the car lights)		⓪—①—②—③	R
24	Difficulty recognising traffic signs		⓪—①—②—③	C
25	Difficulty driving in familiar places		⓪—①—②—③	R
26	Difficulty driving in new places		⓪—①—②—③	R
27	Difficulty using landmarks when walking to familiar places		⓪—①—②—③	R
28	Difficulty using landmarks when walking to unfamiliar places		⓪—①—②—③	R
Total				

**Domestic Chores**				

29	Difficulty operating well-known or previously used electric appliances		⓪—①—②—③	C
30	Forgets the location of electric appliances at home		⓪—①—②—③	R
31	Uses the wrong kitchen utensils when cooking		⓪—①—②—③	R
32	Forgets the name of ingredients when cooking		⓪—①—②—③	R
Total				

**Work and Social Life**				

33	Forgets the name(s) of people in work or social situations		⓪—①—②—③	R
34	Forgets the date and time of tasks and events		⓪—①—②—③	R
35	Difficulty allocating priority to tasks and remembering priorities		⓪—①—②—③	R
36	Difficulty getting well dressed and organised to go to meetings		⓪—①—②—③	R
37	Forgets activities in normal daily routine		⓪—①—②—③	C
38	Difficulty recognising friends with whom interacts regularly		⓪—①—②—③	C
Total				

**Health and Lifestyle**				

39	Difficulty recognising medication(s) (e.g., what a certain pill is for)		⓪—①—②—③	C
40	Forgets where medicines are kept		⓪—①—②—③	R
41	Difficulty recognising changes in own physical status (weight, energy)		⓪—①—②—③	C
42	Forgets regular past-time activities (walks, reading)		⓪—①—②—③	C
43	Withdrawn from habitual leisure		⓪—①—②—③	C
Total				
Grand total				

## Data Availability

The data that support the findings of this study are available from the corresponding author upon reasonable request.

## Appendix A: Details of the DoFEL and DoFEL-R Items

We have included the following in the appendix: (i) the original DoFEL items, with those retained highlighted in green, and (ii) the revised DoFEL measure.

Total Relational Functions (R) (Total 24) ____ out of _____.

Total Conjunctive Functions (C) (Total 20) ____ out of _____.

First, to calculate standardised scores, exclude items are marked as ‘not applicable'. We add up the responses for the remaining items to get the total score. We count the included items and multiply by 3 to find the maximum score. We divide the total score by the maximum score to get a standardised score between 0 and 1. Higher scores indicate greater impairment in daily living functions. We are working on developing cut-off points to indicate differing levels of impairment.

## Supporting Information

Section 1: Demographic Information for Step 1 of the Analysis.

Section 2: Prior Assumption Testing for Step 1 of the Analysis.

Section 3: Factor Loadings and Communalities for Step 1 of the Analysis.

Section 4: Ratings provided by Dementia Professionals for Step 2 of the Analysis.

Section 5: Prior Assumption Testing for Step 3 of the Analysis.

Section 6: Neuropsychological Data from Step 3 of the Analysis.
