# Supplementary material for: Examining the Cognitive Underpinnings of Functional Decline in Prodromal Alzheimer's Disease: Insights From the Details of Functions of Everyday Life (DoFEL) Scale
Source: J Aging Res. 2025 Aug 12;2025:2610700. doi: 10.1155/jare/2610700 (PMC12364600; doi:10.1155/jare/2610700)
Supplement: Supporting Information — Additional supporting information can be found online in the Supporting Information section. [file 2610700.f1.docx]

**Supplementary Material**

Section 1

The table below provides demographic information for the sample used in Step 1.

| ***Characteristic*** | ***Total Sample*** |
| --- | --- |
| Mean age (years) | 70.5 |
| Median age (years) | 69 |
| Mean educational attainment level (years) | 15.8 |
| Gender (% female) | 54.4 |
| *Supplementary Material Table 1: Demographic characteristics for the sample in Step 1* | |

Section 2

The table below outlines the results of the prior assumption testing for the principal axis factoring used in Step 1.

| **DoFEL Subscale** | **Determinant Score of The Correlation Matrix** | **Overall Kaiser-Meyer-Olkin Measure** | **Bartlett’s Test of Sphericity** |
| --- | --- | --- | --- |
| *Shopping and Money* | > 0.00001 | 0.87 | *p* < 0.001 |
| *Objects and People* | > 0.00001 | 0.78 | *p* < 0.001 |
| *Technology and Communication* | > 0.00001 | 0.92 | *p* < 0.001 |
| *Transport and Mobility* | > 0.00001 | 0.93 | *p* < 0.001 |
| *Domestic Chores* | > 0.00001 | 0.52 | *p* < 0.001 |
| *Work and Social Life* | > 0.00001 | 0.81 | *p* < 0.001 |
| *Health and Lifestyle* | > 0.00001 | 0.79 | *p* < 0.001 |
| **Supplementary Table 2:** Prior assumption testing to determine the suitability for PAF on DoFEL subscales.  *The Determinant Score of the Correlation Matrix was used to identify an absence of multicollinearity, as indicated by > 0.00001; The Kaiser-Meyer-Olkin (KMO) Measure of Sampling Adequacy was used to indicate the proportion of variance in the DoFEL subscales that might be influenced by underlying factors. Higher values (close to 1.0) generally indicate that factor analysis may be useful; Bartlett’s Test of Sphericity was employed to assess the likelihood of observing significant correlations within the correlation matrix of DoFEL subscales, indicating potential interrelationships among variables.* | | | |

Section 3

The following seven tables show the factor loadings and communalities values for each DoFEL subscale.

*The Shopping and Money Subscale*

| *DoFEL Items* | *Rotated Factor Coefficients* | | *Communalities* |
| --- | --- | --- | --- |
|  | Factor 1 | Factor 2 |  |
| Difficulty handling money when paying or receiving change (Relational) | 0.96 |  | 0.729 |
| Misplacing money or forgetting where to find it (Relational) | 0.77 |  | 0.672 |
| Forgets the location of products at home or in supermarkets (Relational) | 0.65 |  | 0.535 |
| Difficulty remembering items without a shopping list (Conjunctive) |  | 0.86 | 0.501 |
| Difficulty recognising common products in supermarkets or shops (Conjunctive) |  | 0.55 | 0.780 |
| *Supplementary Table 3: Loadings of the PAF Rotated Structure Matrix of the Shopping and Money Subscale with Communalities* | | | |

*The Objects and People Subscale*

| *DoFEL Items* | *Rotated Factor Coefficients* | | *Communalities* |
| --- | --- | --- | --- |
|  | Factor 1 | Factor 2 |  |
| Difficulty recognising familiar items (Conjunctive) | 0.80 | -0.61 | 0.872 |
| Difficulty recognising personal items (Conjunctive) | 0.79 | -0.59 | 0.843 |
| Difficulty recognising own car (Conjunctive) | 0.76 | -0.50 | 0.702 |
| Difficulty recognising faces of well-known people (Conjunctive) | 0.54 |  | 0.394 |
| Difficulty recognising the house of new friends (Conjunctive) | 0.48 |  | 0.435 |
| Difficulty remembering the location of personal items (Relational) |  | 0.62 | 0.443 |
| Difficulty recalling the name of people recently met (Relational) |  | 0.59 | 0.391 |
| Forgetting where the car was parked (Relational) |  | 0.56 | 0.483 |
| Difficulty recalling the name of well-known people (Relational) |  | 0.55 | 0.417 |
| *Supplementary Table 4: Loadings of the PAF Rotated Structure Matrix of the Objects and People Subscale with Communalities* | | | |

*The Technology and Communication Subscale*

| *DoFEL Items* | *Rotated Factor Coefficients* | | *Communalities* |
| --- | --- | --- | --- |
|  | Factor 1 | Factor 2 |  |
| Difficulty recognising and operating online communication by email (Relational) | -0.93 |  | 0.878 |
| Difficulty dialling using landline phones (Conjunctive) | 0.92 |  | 0.837 |
| Difficulty dialling using mobile phones (Conjunctive) | 0.91 |  | 0.804 |
| Difficulty recognising own mobile phone (Conjunctive) | 0.89 |  | 0.793 |
| Difficulty recognising own mobile phones ringtone (Conjunctive) | 0.84 |  | 0.728 |
| Difficulty operating the cash machine (Relational) | -0.79 |  | 0.786 |
| Difficulty recalling the pin code of different bank cards (Relational) | -0.63 |  | 0.457 |
| Forgets recently learned telephone numbers (Conjunctive) | 0.60 |  | 0.411 |
| Difficulty keeping track of storylines in TV programs (Relational) | -0.59 | 0.48 | 0.460 |
| *Supplementary Table 5: Loadings of the PAF Rotated Structure Matrix of the Technology and Communication Subscale with Communalities* | | | |

*The Transport and Mobility Subscale*

| *DoFEL Items* | *Rotated Factor Coefficients* | *Communalities* |
| --- | --- | --- |
|  | Factor 1 |  |
| Difficulty using landmarks when walking to familiar places (Relational) | 0.91 | 0.810 |
| Forgetting basic actions when driving (Relational) | 0.89 | 0.811 |
| Difficulty driving in new places (Relational) | 0.89 | 0.773 |
| Difficulty driving in familiar places (Relational) | 0.88 | 0.773 |
| Difficulty using landmarks when walking to unfamiliar places (Relational) | 0.86 | 0.727 |
| Difficulty recognising traffic signs (Conjunctive) | -0.69 | 0.513 |
| *Supplementary Table 6: Loadings of the PAF Rotated Structure Matrix of the Transport and Mobility Subscale with Communalities* | | |

*The Domestic Chores Subscale*

| *DoFEL Items* | *Rotated Factor Coefficients* | | *Communalities* |
| --- | --- | --- | --- |
|  | Factor 1 | Factor 2 |  |
| Difficulty operating well-known and previously used electric appliances (Conjunctive) | 0.82 |  | 0.560 |
| Forgetting the name of ingredients when cooking (Relational) | -0.79 |  | 0.522 |
| Forgetting the location of electric appliances at home (Relational) |  | 0.73 | 0.410 |
| Using the wrong kitchen utensils when cooking (Relational) |  | 0.73 | 0.405 |
| *Supplementary Table 7: Loadings of the PAF Rotated Structure Matrix of the Shopping and Money Subscale with Communalities* | | | |

*The Work and Social Life Subscale*

| *DoFEL Items* | *Rotated Factor Coefficients* | | *Communalities* |
| --- | --- | --- | --- |
|  | Factor 1 | Factor 2 |  |
| Difficulty allocating priority to tasks and remembering these (Relational) | 0.85 |  | 0.704 |
| Forgets the name of people at work (Relational) | 0.84 |  | 0.706 |
| Forgets the date and time of tasks at work (Relational) | 0.81 |  | 0.728 |
| Difficulty recognising friends with whom interacts regularly (Conjunctive) | -0.79 |  | 0.682 |
| Being well dressed and groomed to attend work and social meetings (Relational) | 0.78 |  | 0.589 |
| Forgets daily work routines (Conjunctive) | -0.71 | 0.49 | 0.660 |
| *Supplementary Table 8: Loadings of the PAF Rotated Structure Matrix of the Work and Social Life Subscale with Communalities* | | | |

*The Health and Lifestyle Subscale*

| *DoFEL Items* | *Rotated Factor Coefficients* | | *Communalities* |
| --- | --- | --- | --- |
|  | Factor 1 | Factor 2 |  |
| Difficulty recognising medication and what it is for (Conjunctive) | 0.96 |  | 0.884 |
| Difficulty recognising changes in own physical health status (Conjunctive) | 0.93 |  | 0.868 |
| Becoming more withdrawn from habitual leisure activities (Conjunctive) | 0.93 |  | 0.837 |
| Forgets regular pastime activities (Conjunctive) | 0.90 |  | 0.798 |
| Forgets where medicines are kept (Relational) |  | 0.58 | 0.407 |
| *Supplementary Table 9: Loadings of the PAF Rotated Structure Matrix of the Health and Lifestyle Subscale with Communalities* | | | |

Section 4

The table below shows the item classifications per the research team and those as classified by the factorial analysis, whereby ‘R’ = relational binding, ‘C’ = conjunctive binding, and ‘M’ = mixed (where both types of binding were seemingly represented). Also included in the table are the content validity ratings for each item from Step 2 of the study.

| **Item** | **Item Classification by Research Team** | **Item Classification through Factorial Analysis** | **Item Classification by Dementia Professionals** |
| --- | --- | --- | --- |
| Misplacing money or forgetting where to find it | R | R | SA |
| Forgets the location of products at home or in supermarkets | R | R | SA |
| Difficulty remembering the location of personal items | R | R | SA |
| Difficulty recalling the pin code of different bank cards | R | M | SA |
| Forgets where medicines are kept | R | R | SA |
| Difficulty recalling the name of people recently met | R | R | SA |
| Forgetting where the car was parked | R | R | SA |
| Difficulty recalling the name of well-known people | R | R | SA |
| Difficulty keeping track of storylines in TV programs | R | R | SA |
| Difficulty using landmarks when walking to familiar places | R | R | SA |
| Difficulty driving in familiar places | R | R | SA |
| Difficulty driving in new places | R | R | WA |
| Difficulty using landmarks when walking to unfamiliar places | R | R | WA |
| Forgetting the location of electric appliances at home | R | R | WA |
| Difficulty handling money when paying or receiving change | R | R | WA |
| Forgetting the name of ingredients when cooking | R | M | WA |
| Forgets the name of people at work | R | R | WA |
| Difficulty recognising and operating online communication by email | R | M | NR |
| Difficulty operating the cash machine | R | M | NR |
| Difficulty allocating priority to tasks and remembering these | R | R | NR |
| Forgets the date and time of tasks at work | R | R | NR |
| Using the wrong kitchen utensils when cooking | R | R | NR |
| Being well dressed and groomed to attend work and social events | R | R | NR |
| Forgetting basic actions while driving | R | R | NR |
| Difficulty recognising own mobile phone | C | C | SA |
| Difficulty in recognising common products in supermarkets or shops | C | C | SA |
| Difficulty remembering items without a shopping list | C | C | SA |
| Difficulty recognising familiar items | C | M | SA |
| Difficulty recognising own car | C | M | SA |
| Difficulty recognising the house of new friends | C | C | SA |
| Difficulty recognising medication and what it is for | C | C | SA |
| Difficulty recognising faces of well-known people | C | C | WA |
| Difficulty recognising traffic signs | C | M | WA |
| Difficulty recognising friends with whom interacts regularly | C | C | WA |
| Difficulty recognising personal items | C | M | WA |
| Difficulty recognising own mobile phones ringtone | C | C | WA |
| Forgets regular pastime activities | C | C | NR |
| Difficulty dialling using landline phones | C | C | NR |
| Forgets recently learnt telephone numbers | C | C | NR |
| Difficulty operating well-known and previously used electric appliances | C | C | NR |
| Difficulty recognising changes in own physical health status | C | C | NR |
| Difficulty dialling using mobile phones | C | C | NR |
| Forgets daily work routines | C | M | NR |
| Becoming more withdrawn from habitual leisure activities | C | M | NR |
| *Supplementary Table 10: Details of factor analytic and content validity index classifications for binding*  **Item Classification for Binding:** ‘R’ = Relational; ‘C’ = Conjunctive; ‘M’ = Mixed  **Item Relevance to Binding:** ‘SA’ Strong Agreement; ‘WA’ = Weaker Agreement; ‘NR’ = Not Relevant  *Factorial Analysis Classification: Items with minimum commonalities (h^2^) scores greater than 0.4 were used to decide the classification without evidence of cross-loading.  *Dementia Professionals Classification: Items with an individual CVI of 0.75 and greater were seen to have a strong agreement; those with 0.5 to 0.25 were seen as having a weak agreement, and less than 0.25 were seen as non-relevant to binding. | | | |

Section 5

The table below outlines the results of prior assumption testing conducted for the Confirmatory Factor Analysis.

| **DoFEL Subscale** | **Bartlett’s Test of Sphericity** | **Kaiser-Mayer-Olkin Measure of Sampling Adequacy** |
| --- | --- | --- |
| *Shopping and Money* | *p* < 0.001 | 0.76 |
| *Objects and People* | *p* < 0.001 | 0.66 |
| *Technology and Communication* | *p* < 0.001 | 0.81 |
| *Transport and Mobility* | *p* < 0.001 | 0.72 |
| *Domestic Chores* | *p* < 0.001 | 0.86 |
| *Work and Social Life* | *p* < 0.001 | 0.61 |
| *Health and Lifestyle* | *p* < 0.001 | 0.55 |
| **Supplementary Table 11:** Prior assumption testing to determine the suitability for CFA on DoFEL subscales. | | |

Section 6

The table below provides data on the overall neuropsychological test performance for the sample used in Step 3 of the analysis. This has been provided by group membership, either MCI or healthy older adults.

| *Neuropsychological Test* | *Mean Score* | |
| --- | --- | --- |
|  | MCI | HOA |
| ACE-R | 78.5 | 96.1 |
| Original DoFEL (raw score) | 83.1 | 71.3 |
| Original DoFEL (standardised score) | 0.41 | 0.32 |
| Revised DoFEL (raw score) | 66.5 | 34.8 |
| Revised DoFEL (standardised score) | 0.59 | 0.24 |
| VSTBMT | 0.66 | 0.87 |
| *Supplementary Table 12: Cognitive and functional data by diagnostic group* | | |
